# Susceptibility of caprine mastitis pathogens to tildipirosin, gamithromycin, oxytetracycline, and danofloxacin: effect of serum on the in vitro potency of current macrolides

**DOI:** 10.1007/s11274-022-03407-0

**Published:** 2022-09-13

**Authors:** Juan Sebastian Galecio, Elisa Escudero, Juan Carlos Corrales, Edgar García-Romero, Christian de la Fe, Verónica Hernandis, Pedro Marin

**Affiliations:** 1 Department of Pharmacology, Faculty of Veterinary Medicine, University of Murcia, 30100 Murcia, Spain; 2 Escuela de Medicina Veterinaria, Colegio de Ciencias de La Salud, Universidad San Francisco de Quito, Campus Cumbayá, Quito, EC 170157 Ecuador; 3 Ruminant Health Research Group, Department of Animal Health, Faculty of Veterinary Sciences, Regional Campus of International Excellence “Campus Mare Nostrum”, University of Murcia, 30100 Murcia, Spain

**Keywords:** Tildipirosin, Gamithromycin, Oxytetracycline, Danofloxacin, Mastitis, MIC, *Mycoplasma agalactiae*, Serum, Goats

## Abstract

Mastitis is a significant disease in dairy ruminants, causing economic losses to the livestock industry and severe risks to public health. Antibiotic therapy is one of the most crucial practices to treat mastitis, although the susceptibility of caprine mastitis pathogens to current antibiotics has not been tested under standard or modified incubation conditions. This work evaluated the in vitro activity of tildipirosin, gamithromycin, oxytetracycline, and danofloxacin against caprine mastitis pathogens incubated following standard conditions of Clinical and Laboratory Standards Institute (CLSI) and deviation method by 25% supplementation with goat serum. *Mycoplasma agalactiae, Escherichia coli, Staphylococcus aureus*, *Streptococcus* spp., and coagulase-negative Staphylococci (CNS) were isolated from dairy goats with mastitis in Spain. Minimum inhibitory concentrations (MICs) were determined using the broth microdilution technique. The lowest MIC_90_ under standard conditions was obtained with danofloxacin for mastitis-causing pathogens. An exception was *M. agalactiae*, where danofloxacin and oxytetracycline obtained low values. However, after adding serum, gamithromycin showed the lowest MIC_50_ for *S. aureus*, *Streptococcus* spp., and CNS. The lowest MIC_50_ was obtained with all the antibiotics tested (< 0.125 µg/ml) against *M. agalactiae*. Supplementing with serum resulted in a significant variation in tildipirosin and gamithromycin MIC values for CNS, *S. aureus*, *M. agalagtiae*, and *E. coli*. In brief, the MIC for antibiotics used against mastitis should be determined under conditions closely resembling intramammary infections to obtain representative susceptibility patterns against mastitis pathogens. Caprine mastitis pathogens were broadly susceptible to danofloxacin under standard conditions. The potency of macrolides against caprine mastitis pathogens increases when serum is present in culture media.

## Introduction

Mastitis is considered one of the most important diseases in dairy animals and frequently occurs due to the colonization of fungi, viruses, or bacteria, especially if poor milking management and unhygienic conditions are present in dairy farms (Ruegg 2017). Mammary gland inflammation is classified into clinical and subclinical infections. Subclinical mastitis is the most common presentation in goats (Persson and Olofsson 2011), where milk appears normal with no apparent abnormalities in mammary tissues. However, the subclinical form could lead to clinical form or establish a reservoir of pathogens that acts as a source of infection to healthy animals. In terms of livestock production, mastitis can result in relevant economic losses owing to reduced milk yields, poor quality of the milk, early elimination of animals, and increased treatment costs (Gelasakis et al. 2015).

Staphylococci are the most common pathogen group present in mastitis (Virdis et al. 2010), where *Staphylococcus aureus* has been associated with clinical mastitis. In contrast, coagulase-negative Staphylococci (CNS) are considered the most prevalent in subclinical mastitis in dairy goats (Mishra et al. 2014). *Streptococcus* spp*.,* is reported to be the most common cause of clinical and subclinical mastitis in goats after Staphylococci (Bergonier et al. 2013). Other isolated pathogens in goat mastitis include *Mannhemia haemolytica, Escherichia coli, Clostridium perfringens, Mycoplasma* spp*., Pseudomonas* and *Nocardia* species (Olechnowicz and Jaśkowski 2014).

*Mycoplasma* spp*.* is the causative intracellular agent of contagious agalactia, a multi-aetiological syndrome that generates significant economic losses, principally in Mediterranean countries with small ruminant dairy industries (Gómez-Martín et al. 2013). Four different species have been associated with this disease: *Mycoplasma agalactiae, Mycoplasma mycoides subsp. capri, Mycoplasma capricolum subsp. capricolum* and *Mycoplasma putrefaciens*. Nevertheless, *M. agalactiae* is the most frequent agent isolated in sheep and goats. In endemic areas, the typical presentation of the disease is subclinical mastitis, with evolution to clinical mastitis in some animals. Clinical disease not only affects the mammary gland but can also be associated with arthritis, keratoconjunctivitis, septicaemia, pneumonia, and abortions (Bergonier et al. 2013; Gómez-Martín et al. 2013). Currently, tetracyclines, macrolides, and fluoroquinolones are widely recommended drugs against contagious agalactia (Gómez-Martín et al. 2013). Unfortunately, their current use and bacteriological/clinical outcomes are rarely reported. A few antibiotics are marketed explicitly for use in small ruminants; however, due to such circumstances, products authorized in cattle are used to treat contagious agalactia, rationalized on the cascade principle. Antibiotics provide clinical recovery in this disease but infrequently complete bacteriological cure (Bergonier et al. 1997), as was shown recently in two studies where long-acting oxytetracycline and macrolides were used (Agnello, et al. 2012; Giadinis et al. 2008). *Mycoplasma bovis*, another mastitis pathogen closely related to *M. agalactiae*, has been found to increase its antibiotic resistance level for almost all antimicrobials, except for fluoroquinolones, in contemporary bacterial isolates (Gautier-Bouchardon et al. 2014). In these circumstances, updated antibiotic susceptibility of *M. agalactiae* is essential. It has only been addressed within scarce studies (Poumarat et al. 2016).

Moreover, some experiments suggest that the antimicrobial activity of some macrolides may be undervalued if we only consider the MIC obtained by CLSI methods. For example, a study showed that tildipirosin MIC against *Actinobacillus pleuropneumoniae* incubated on standard methods was higher than those obtained by adding serum in different proportions to the culture medium (5%, 10%, 25%, and 50%) (Rose et al. 2013). For tulathromycin, calf serum has been reported to improve the control of pH media, consequently enhancing MIC determinations. Moreover, a buffer capacity has been attributed to plasma proteins, which are essential serum components (Lees et al. 2017), and low molecular weight proteinaceous components of the serum interact with azithromycin and other macrolides, such as roxithromycin and erythromycin, enhancing its antibacterial activities (Pruul and McDonald 1992). Therefore, the determination of macrolide dosages for therapeutic use should be derived from pharmacodynamic data obtained from biological fluids because in vitro measurement of MIC in broth, performed following international recommended methods, may be misleading for estimating the in vivo potency of these antibiotics. Thus, the objective of the present study was to evaluate the in vitro activity of tildipirosin, gamithromycin, oxytetracycline, and danofloxacin against *M. agalactiae*, *E. coli*, and *S. aureus, Streptococcus* spp., and CNS incubated on CLSI conditions and deviation from CLSI methods by 25% supplementation with goat serum.

## Materials and methods

### Isolation and identification of pathogens

The isolates included in the current study were obtained from the strain collection of the University of Murcia—Spain. The examined mastitis pathogens were isolated from individual mastitis samples in goat flocks during 2018 and 2020 in the southeastern region of Spain. Altogether, 107 isolates were included in the study, subdivided into 39 CNS, 37 *S. aureus*, 11 *Streptococcus* spp*.*, 10 *E. coli*, and 10 *M. agalactiae*.

Ten microliters of each sample was spread onto the surface of blood agar plates, incubated aerobically at 37 °C and examined after 24, 48 and 72 h. A subclinical intramammary infection isolation threshold was established in five identical colonies (500 cfu/ml).

The identification of Gram-positive, catalase-positive cocci was performed according to the presence or absence of target hemolysis. For isolates with target hemolysis, a commercial latex agglutination kit for the identification and differentiation of *S. aureus* (Staphytec Plus, Oxoid, Basingstoke, UK) were used. Isolates without target hemolysis were inoculated into API Staph strips (bioMérieux) for identification. Gram-positive, catalase-negative cocci were identified as *Streptococcus* spp*.* To investigate the presence of *Streptococcus agalactiae*, the CAMP test, esculin hydrolysis and hemolysis were considered.

Oxidase tests were performed for Gram-negative bacilli. If the oxidase test was negative, isolates were inoculated on MacConkey agar and investigated for indole production, utilization of citrate as the sole source of carbon, methyl red test and Voges-Proskauer test. Isolates lactose positive on MacConkey agar, indole positive, citrate negative, methyl red positive and Voges-Proskauer negative were identified as *E. coli*.

For the isolation of *M. agalactiae*, solid and liquid pH media were used (Kirchhoff and Rosengarten, 1984). Isolates from previously cloned single colonies of *M. agalactiae* were identified by PCR (Marenda et al. 2005).

### Antimicrobial susceptibility testing

Tildipirosin, gamithromycin, oxytetracycline, and danofloxacin (Cymit Química, Barcelona, Spain) were selected for the study. Antibiotics were dissolved in suitable solvents to make stock solutions and then diluted in sterile distilled water following the guidelines of the Clinical and Laboratory Standards Institute (2009). Blood samples were obtained from ten healthy female Murciano-Granadina goats aged 3–5 years. The samples obtained were centrifuged at 1500×*g* for 10 min, and the freshly collected serum was pooled and divided into 1-ml portions, stored at −80 °C, and thawed immediately before the experiment.

### Antimicrobial susceptibility testing for CNS, *S. aureus*, *Streptococcus* spp., and *E. coli*

Standard conditions or modifications from CLSI methods by 25% goat serum supplementation were performed to determine the MIC of tildipirosin, gamithromycin, oxytetracycline, and danofloxacin. Minimum inhibitory concentration tests were performed by the microdilution broth technique (Clinical and Laboratory Standards Institute 2009) using U-bottom 96-well microtiter plates. Serial two-fold dilutions of the antimicrobial agents were prepared starting from the stock solution. Broth dilutions were made using Mueller–Hinton broth (MHB) (Merck, Madrid, Spain) for CNS, *S. aureus*, and *E. coli*. To investigate *Streptococcus* spp., cation-adjusted Mueller–Hinton broth (Merck, Madrid, Spain) with 5% defibrinated horse blood (Thermo Fisher Scientific, Massachusetts, USA) was used. Concentrations of all antibiotics ranging from 0.03 to 128 mg/l were used. Inocula were prepared by diluting an overnight MHB culture in buffered saline solution to a density of 0.5 on the McFarland Turbidity Scale and finally diluting again 40-fold before testing. The U-bottomed microtiter plates were incubated at 37 °C and observed 24 h later. The MIC was defined as the lowest concentration of antibiotic at which bacterial growth was completely inhibited. The reference strains *S. aureus* (ATCC 29213) and *E. coli* (ATCC 25922) were used as controls.

### Antimicrobial susceptibility testing for *M. agalactiae*

The minimal inhibitory concentration was determined according to the recommendations of Hannan (2000). Briefly, a stationary-phase culture of each isolate was carried out in mycoplasma medium without antimicrobials supplemented with phenol red (0.005%) in 96-well round-bottomed plates. Each antibiotic was added to achieve each of the pre-established final concentrations (from 32 to 0.006 μg/ml) and a final concentration of the mycoplasma cultures of 10^3^–10^5^ colour-changing units/ml. Positive (lacking antibiotic) and negative (lacking mycoplasmas) controls were also added. The plates were then sealed and incubated at 37 °C. After 48 h, the plates were examined for colour change. The MIC was defined as the lowest concentration of each antibiotic at which no *M. agalactiae* growth (no colour change) was observed.

### Statistical analysis

Normality of the data was investigated using the Shapiro–Wilk test. The antibacterial susceptibility of isolates incubated in standard conditions was contrasted with 25% goat serum addition to culture media. Susceptibility differences between both methods (CLSI conditions and deviation from CLSI methods by 25% supplementation with goat serum) were determined by the Wilcoxon rank-sum test, and a significant difference was considered when p < 0.05. Statistical analysis was performed with IBM SPSS Statistics 24 (New York, NY, USA).

Susceptibility of CNS, *S. aureus, Streptococcus* spp*., E. coli*, and *M. agalactiae* are presented as minimal concentrations of tildipirosin, gamithromycin, oxytetracycline, and danofloxacin that inhibited 50% and 90% of these isolates (MIC_50_ and MIC_90_).

The percentage of CNS, *S. aureus, and Streptococcus* spp., inhibited by increasing concentrations of tildipirosin, gamithromycin, and oxytetracycline have a bimodal distribution, confirming that the data were plotted using ggplot2 [R version 4.0.4 (2021-02-15)].

## Results

**Fig. 1:**
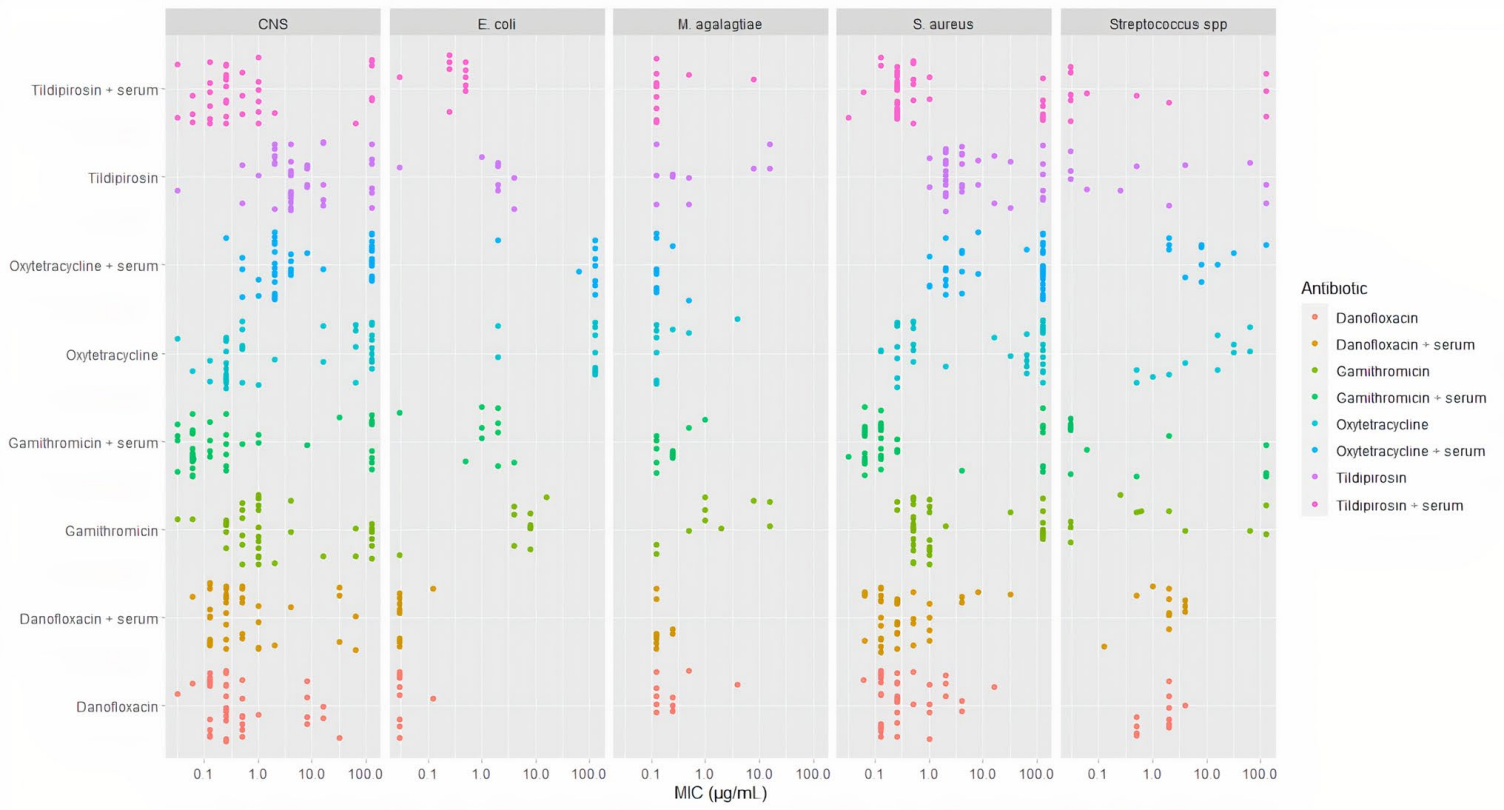
Minimum inhibitory concentration (µg/ml) of tildipirosin, gamithromycin, danofloxacin and oxytetracycline on CNS, *S. aureus, Streptococcus* spp*., E. coli*, and *M. agalactiae* strains isolated from mastitic goat milk incubated in Mueller Hinton broth and Mueller Hinton broth supplemented with 25% goat serum

Modifying the culture media by adding goat serum resulted in appreciable variation of the MIC of tildipirosin, gamithromycin, and oxytetracycline for *S. aureus*, CNS, and *M. agalagtiae*, but MIC values were unchanged or slightly increased for danofloxacin (Fig. 1).

The MIC_50_ and MIC_90_ of tildipirosin, gamithromycin, oxytetracycline, and danofloxacin are presented in Table 1.

**Table 1:** Minimum inhibitory concentration values (µg/ml) of tildipirosin, gamithromycin, danofloxacin and oxytetracycline that inhibited 50% and 90% of the isolates (MIC_50_ and MIC_90_, respectively) on CNS, *S. aureus, Streptococcus* spp*.*, *E. coli* and *M. agalactiae* strains isolated from mastitic goat milk incubated in Mueller Hinton Broth and Mueller Hinton Broth supplemented with 25% goat serum

Strains	Tildipirosin				Gamithromycin				Danofloxacin				Oxytetracycline			
	MBH		MBH + 25%		MBH		MBH + 25%		MBH		MBH + 25%		MBH		MBH + 25%	
	MIC_50_	MIC_90_	MIC_50_	MIC_90_	MIC_50_	MIC_90_	MIC_50_	MIC_90_	MIC_50_	MIC_90_	MIC_50_	MIC_90_	MIC_50_	MIC_90_	MIC_50_	MIC_90_
CNS (n = 39)	4	> 128	0.25	> 128	1	> 128	0.125	> 128	0.25	8	0.25	32	0.5	> 128	4	> 128
*Staphylococcus aureus* (n = 37)	4	> 128	0.25	> 128	1	> 128	0.125	> 128	0.25	2	0.25	4	64	128	128	> 128
*Streptococcus* spp (n = 11)	4	> 128	0.5	> 128	0.5	> 128	0.06	> 128	2	2	2	4	16	64	8	32
*Escherichia coli* (n = 10)	2	4	0.25	0.5	8	8	1	2	< 0.03	< 0.03	< 0.03	< 0.03	> 128	> 128	> 128	> 128
*Mycoplasma agalactiae* (n = 10)	0.25	> 16	< 0.125	0.5	1	16	< 0.125	0.5	< 0.125	0.5	< 0.125	0.25	< 0.125	0.5	< 0.125	0.25

*MHB* Mueller Hinton Broth, *MHB* + 25% Mueller Hinton Broth supplemented with 25% goat serum, *CNS* coagulase-negative Staphylococci

Gamithromycin and tildipirosin showed a reduction of the MIC_50_ when the culture media was supplemented with goat serum for CNS and *S. aureus*. On the other hand, the MIC_50_ of oxytetracycline increased by 3 log_2_ dilution when goat serum was added for CNS and *S. aureus*. The lowest MIC_50_ and MIC_90_ values were obtained with danofloxacin under standard conditions and deviation from the CLSI method (MIC_50_ = 0.25 µg/ml for CNS and *S. aureus*; MIC_90_ = 8–32 µg/ml for CNS and 2–4 µg/ml for *S. aureus*).

Supplementing with serum the culture media caused a reduction of the MIC_90_ of both gamithromycin and tildipirosin by approximately 5 log_2_ dilution, resulting in a significant decrease of the MIC_90_ (> 16 µg/ml) under standard conditions when compared to supplementation with goat serum (0.5 µg/ml) for *M agalactiae*. In addition, the MIC_50_ and MIC_90_ of tildipirosin and gamithromycin were higher than those obtained with danofloxacin and oxytetracycline under standard conditions for *M agalactiae*. Nevertheless, after modifying the culture medium conditions with serum, similar MIC_50_ and MIC_90_ values were obtained for gamithromycin, tildipirosin, danofloxacin, and oxytetracycline.

Danofloxacin showed the lowest MIC_50_ and MIC_90_ values against *E. coli* both under standard conditions and after adding goat serum. The supplementation with serum resulted in a decrease in MIC_90_ by 3 log_2_ dilution for tildipirosin and by 2 log_2_ dilution for gamithromycin (p < 0.05).

Supplementing the culture media with serum did not cause a variation in the MIC_50_ and MIC_90_ of gamithromycin, tildipirosin, danofloxacin, and oxytetracycline for *Streptococcus* spp. (p > 0.05)*.* The lowest MIC_50_ and MIC_90_ for *Streptococcus* spp*.* were obtained with gamithromycin and danofloxacin, respectively.

## Discussion

Contagious agalactia results in significant economic losses for farmers due to decreased milk yields, abortions, reduced growth rates, early culling in affected animals, and the expenses needed for control or treatment measures. Moreover, the impact of Mycoplasmas on milk quality is probably underestimated (Al-Farha et al. 2017; Contreras et al. 2008). Antimicrobial therapy is one of the most critical practices to control this syndrome but requires a withdrawal period resulting in significant milk production losses. Fluoroquinolones, macrolides, and tetracyclines are used as standard treatments for controlling contagious agalactia (Bergonier et al. 1997; Gómez-Martín et al. 2013). In this study, danofloxacin and oxytetracycline were the most effective antibiotics, with an MIC_90_ of 0.5 µg/ml. These values are in agreement with previously reported data (Antunes et al. 2008; Garnica et al. 2013). After adding 25% goat serum, similar values were obtained with the four antibiotics for *M. agalactiae* (MIC_90_ of tildipirosin and gamithromycin = 0.5 µg/ml; MIC_90_ of danofloxacin and oxytetracycline = 0.25 µg/ml). These results suggest that current macrolides may be helpful to treat contagious agalactia since they are potent weak bases that are ion-trapped within acidic intracellular compartments, such as lysosomes and phagosomes. A beneficial consequence of macrolide intracellular accumulation is its increased activity against intracellular pathogens (Ahmad et al. 2010; Fietta et al. 1997). In goats, tildipirosin concentrations in somatic cells following subcutaneous and intramuscular administrations were 22–27 times higher than simultaneous plasma concentrations (Galecio et al. 2022). It is crucial to know which antibiotics of veterinary use are effective against *M. agalactiae* isolated from goats to successfully treat contagious agalactia, especially in endemic areas such as Spain and neighboring Mediterranean countries. It is well known that the in vitro sensitivity of antibiotics is not always consistent with treatment effectiveness in field conditions, but antibiotics with high MIC values against these microorganisms are likely to be ineffective in successfully treating sick animals (Barlow 2011; Constable and Morin 2003). Staphylococci are the most common pathogen species isolated in dairy goats affected with subclinical mastitis, causing subclinical to clinical mastitis, with various presentations from mild to acute toxic forms or even gangrenous mastitis, and are one of the most important causes of discarding dairy animals. Staphylococci grow on udder skin, inside the teat canal, and in mammary tissues and are disseminated through improper and unhygienic milking routines (Ruegg 2017). In the present study, the lowest MIC_90_ values for CNS were obtained with danofloxacin (MIC_90_ = 8 µg/ml), obtaining MIC_90_ values higher than 128 µg/ml for the rest of the antibiotics tested. After adding 25% goat serum, gamithromycin showed a lower MIC50 value than danofloxacin (MIC_50_ of gamithromycin = 0.125 µg/ml). Only scant data are found for MIC against CNS isolated from dairy goats, but the reported MIC_90_ was obtained for oxytetracycline (MIC_90_ = 1 µg/ml), and other fluoroquinolones (MIC_90_ of ofloxacin = 1 µg/ml) were lower than those of our study (Virdis et al. 2010).

*Staphylococcus aureus* is one of the most important etiologic agents in ruminant mastitis. Intramammary infections are complicated to treat and eradicate because resistant strains of *S. aureus* frequently emerge after negligible antibiotic pressure. Strains tested in this study showed high MIC_90_ values against tildipirosin, gamithromycin, and oxytetracycline (MIC_90_ ≥ 128 µg/ml). The most effective antimicrobial tested against *S. aureus* strains isolated from milk was danofloxacin (MIC_90_ = 2 µg/ml). Lower values for danofloxacin have been reported previously [MIC_90_ = 0.25 µg/ml (Serrano-Rodríguez et al. 2017); MIC_90_ = 0.5 µg/ml (Marín et al. 2010)]. After adding 25% goat serum, the susceptibility of tildipirosin and gamithromycin against *S. aureus* was not modified. The reason may be that the MIC_90_ values of the two macrolides under standard conditions were greater than 128 µg/ml, but the exact value was not quantified. Therefore, when adding serum, it is possible that MICs decreased but not enough to be detected; these values were still higher than 128 µg/ml.

The other commonly tested bacteria in the present study were *E. coli*, which is responsible for environmental mastitis in dairy ruminants. Coliforms develop when farmers follow unsanitary housing and impair pre-milking teat disinfection (Ruegg 2017). Danofloxacin showed the lowest MIC_50_ and MIC_90_ for *E. coli* under standard conditions and after adding goat serum. Strains tested in the current study showed high MIC_90_ values for oxytetracycline. Minimum inhibitory concentrations that inhibited 90% of caprine mastitis pathogens obtained with tildipirosin (4 µg/ml) and gamithromycin (8 µg/ml) were high, but after modifying the conditions with serum, MIC values decreased dramatically (MIC_90_ = 0.5 and 2 µg/ml for tildipirosin and gamithromycin, respectively). References are available in the literature about the susceptibility of *E. coli* strains isolated from milk to antimicrobial agents in dairy cows (Shinozuka et al. 2019; Thomas et al. 2015); however, data are unavailable for dairy goats. Similar high MIC_90_ values were found for different tetracyclines in strains isolated from dairy cows (MIC_90_ ≥ 64 µg/ml; Thomas et al., 2015) and lower values for other fluoroquinolones, such as enrofloxacin o marbofloxacin (MIC_90_ = 0.03 and 0.06 µg/ml, respectively; Thomas et al. 2015).

*Streptococcus* spp. is also considered a common etiological cause of clinical and subclinical mastitis in goats after Staphylococci, with *S. agalactiae*, *Streptococcus uberis*, and *Streptococcus dysgalactiae* frequently found in mammary gland infections (Bergonier et al. 2013). As with *E. coli*, there are no published data about the susceptibility of *Streptococcus* spp., isolated from goat mastitis with the antibiotics tested in this study. There are some studies on isolated *Streptococcus* spp. strains from cow mastitis (McDougalla et al. 2013; Thomas et al. 2015) and MIC_90_ values reported to be lower than those in the present study for oxytetracycline (MIC_90_ = 1 and 4 µg/ml for *S. uberis* and *S. dysgalactiae*, respectively; McDougalla et al. 2013), another macrolide (MIC_90_ of tylosin = 2 and 1 µg/ml for *S. uberis* and *S. dysgalactiae*, respectively; Thomas et al. 2015) and different fluoroquinolones, such as enrofloxacin (MIC_90_ = 1 µg/ml for both species; McDougalla et al. 2013) and marbofloxacin (MIC_90_ = 2 µg/ml for *S. uberis*; Thomas et al. 2015). Then, comparisons are difficult to establish.

The present study demonstrates that macrolides, but not danofloxacin and oxytetracycline, have markedly lower MICs against different pathogens when assayed in culture media broth supplemented with serum compared with MHB (CLSI recommendation for in vitro susceptibility testing studies). As antibiotic susceptibility is an essential property to contemplate when assessing its clinical value, these observations open a potentially important expectation concerning the clinical benefits of macrolides in goats affected with mastitis. Artificial growth matrices, such as MHB, are not undoubtedly predictive of bacterial growth in physiological fluids, and as a consequence, they may be poor predictive tools in some cases of antimicrobial drug activity in vivo. Considering these variations between biological fluids and artificial growth media, some authors advocate for the use of physiological fluids in studies of antimicrobial activity testing when the objective is to establish optimal dosing regimens for bacterial killing in vivo (Lees et al. 2017; Nightingale and Murakawa 2012).

Moreover, some buffering capacity has been attributed to proteins, which are major components of serum (Pruul and McDonald 1992; Lees et al. 2017). Antimicrobial activity-enhancing effects of proteinaceous serum components have also been assumed for azithromycin, erythromycin, roxithromycin (Pruul and McDonald 1992) and gamithromicin (Zhou et al 2020). Recent studies have shown that the high susceptibility of *Pasteurella aeruginosa* to macrolides in RPMI 1640 medium (medium used for growing eukaryotic cells) compared to broths may be related to an increase in their accumulation within the bacteria, owing to an alteration of the outer-membrane integrity (caused by the nature of the medium in contact with bacteria) combined with an impairment of their active efflux (decreased expression of oprM) (Buyck et al. 2012). Moreover, tildipirosin and gamithromycin tested in this study, like other macrolides, may have various immunomodulatory benefits that probably contribute to successful clinical outcomes in different infections. Other reported benefits for macrolides may be influenced by enhanced degranulation and apoptosis of neutrophils, enhanced macrophage functions, and its ability to inhibit inflammatory cytokine production (Zarogoulidis et al. 2012).

The effect of milk as a matrix in the culture medium has also been investigated. Some studies have displayed a significant influence on MIC values and have revealed that much of the reduction in antibiotic activity is related to high binding to casein or lipids in milk (Kuang et al. 2009; Owens and Watts 1987). Although macrolides have low protein binding (18–30%) (Papich 2018; Stepanić et al., 2012), incorporating milk from healthy animals into the culture media determines a reduction in the antibacterial activity of erythromycin (Owens and Watts 1987); other milk components probably play an important role. Nevertheless, the milk from infected quarters has broad changes in its composition, with lower casein and lactose content but higher total protein and whey protein concentrations (Forsbäck et al. 2010), which could determine an increased potency of macrolides, as was shown in our study, but further experiments are needed to confirm this assumption.

## Conclusion

Minimal inhibitory concentrations for antibiotics used on mastitis should be determined under conditions closely resembling intramammary infections to obtain representative susceptibility patterns against goat mastitis pathogens. Caprine mastitis pathogens were broadly susceptible to danofloxacin under standard incubation conditions. The potency of macrolides against susceptible caprine mastitis pathogens increases when serum is present in culture media.

## Data Availability

The data that support the findings of this study are openly available in Science Data Bank at 10.11922/sciencedb.01662 published on 2022–04-02.

## References

[CR1] Agnello S, Chetta M, Vicari D (2012). Severe outbreaks of polyarthritis in kids caused by *Mycoplasma mycoides* subspecies capri in Sicily. Vet Rec.

[CR2] Ahmad S, Hunter L, Qin A (2010). Azithromycin effectiveness against intracellular infections of *Francisella*. BMC Microbiol.

[CR3] Al-Farha AA, Hemmatzadeh F, Khazandi M, Hoare A, Petrovski K (2017). Evaluation of effects of Mycoplasma mastitis on milk composition in dairy cattle from South Australia. BMC Vet Res.

[CR4] Antunes NT, Tavío MM, Assuncao P (2008). In vitro susceptibilities of field isolates of *Mycoplasma agalactiae*. Vet J.

[CR5] Barlow J (2011). Mastitis therapy and antimicrobial susceptibility: a multispecies review with a focus on antibiotic treatment of mastitis in dairy cattle. J Mammary Gland Biol Neoplasia.

[CR6] Bergonier D, Berthelot X, Poumarat F (1997). Contagious agalactia of small ruminants: current knowledge concerning epidemiology, diagnosis and control. Rev Sci Tech.

[CR7] Bergonier D, Crémoux RD, Rupp R (2013). Mastitis of dairy small ruminants. Vet Res.

[CR8] Buyck JM, Plésiat P, Traore H (2012). Increased susceptibility of *Pseudomonas aeruginosa* to macrolides and ketolides in eukaryotic cell culture media and biological fluids due to decreased expression of oprM and increased outer-membrane permeability. Clin Infect Dis.

[CR9] Clinical and Laboratory Standards Institute (2009). Methods for dilution antimicrobial susceptibility test for bacteria that grow aerobically; approved standard. NCCLS document M07–A8.

[CR10] Constable PD, Morin DE (2003). Treatment of clinical mastitis. Using antimicrobial susceptibility profiles for treatment decisions. Vet Clin North Am Food Anim Pract.

[CR11] Contreras A, Miranda RE, Sánchez A, De la Fe C, Sierra D, Luengo C, Corrales JC (2008). Presence of *Mycoplasma* species and somatic cell counts in bulk-tank goat milk. Small Rumin Res.

[CR12] Fietta A, Merlini C, Gialdroni GG (1997). Inhibition of intracellular growth of *Staphylococcus aureus* by exposure of infected human monocytes to clarithromycin and azithromycin. J Chemother.

[CR13] Forsbäck L, Lindmark-Månsson H, Andrén A (2010). Evaluation of quality changes in udder quarter milk from cows with low-to-moderate somatic cell counts. Animal.

[CR14] Galecio JS, Marín P, Hernandis V, Botía M, Escudero E (2022). Pharmacokinetics of tildipirosin in plasma, milk, and somatic cells following intravenous, intramuscular, and subcutaneous administration in dairy goats. Pharmaceutics.

[CR15] Garnica ML, Rosales RS, Gonzalo C (2013). Isolation, molecular characterization and antimicrobial susceptibilities of isolates of *Mycoplasma agalactiae* from bulk tank milk in an endemic area of Spain. J Appl Microbiol.

[CR16] Gautier-Bouchardon AV, Ferre S, Le Grand D (2014). Overall decrease in the susceptibility of *Mycoplasma bovis* to antimicrobials over the past 30 years in France. PLoS ONE.

[CR17] Gelasakis AI, Mavrogianni VS, Petridis IG (2015). Mastitis in sheep for the last 10 years and the future of research. Vet Microbiol.

[CR18] Giadinis ND, Petridou EJ, Sofianidis G (2008). Mortality in adult goats attributed to *Mycoplasma capricolum subspecies capricolum*. Vet Rec.

[CR19] Gómez-Martín A, Amores J, Paterna A, De la Fe A (2013). Contagious agalactia due to *Mycoplasma* spp. in small dairy ruminants: epidemiology and prospects for diagnosis and control. Vet J.

[CR20] Hannan PC (2000). Guidelines and recommendations for antimicrobial minimum inhibitory concentration (MIC) testing against veterinary mycoplasma species. International Research Programme on Comparative Mycoplasmology. Vet Res.

[CR21] Kirchhoff H, Rosengarten R (1984). Isolation of a motile mycoplasma from fish. J Gen Microbiol.

[CR22] Kuang Y, Jia H, Miyanaga K, Tanji Y (2009). Effect of milk on antibacterial activity of tetracycline against *Escherichia coli* and *Staphylococcus aureus* isolated from bovine mastitis. Appl Microbiol Biotechnol.

[CR23] Lees P, Illambas J, Potter TJ (2017). A large potentiation effect of serum on the in vitro potency of tulathromycin against *Mannheimia haemolytica* and *Pasteurella multocida*. J Vet Pharmacol Ther.

[CR24] Marenda MS, Sagné E, Poumarat F, Citti C (2005). Suppression subtractive hybridization as a basis to assess *Mycoplasma agalactiae* and *Mycoplasma bovis* genomic diversity and species-specific sequences. Microbiology.

[CR25] Marín P, Escudero E, Fernández-Varón E (2010). Fluoroquinolone susceptibility of *Staphylococcus aureus* strains isolated from caprine clinical mastitis in southeast Spain. J Dairy Sci.

[CR26] McDougalla S, Husseina H, Petrovskib K (2013). Antimicrobial resistance in *Staphylococcus aureus, Streptococcus uberis* and *Streptococcus dysgalactiae* from dairy cows with mastitis. N Z Vet J.

[CR27] Mishra AK, Sharma N, Kumar A (2014). Isolation, characterization and therapeutic potential assessment of bacteriophages virulent to *Staphylococcus aureus* associated with goat mastitis. Iran J Vet Res.

[CR28] Nightingale CH, Murakawa T (2012) Microbiology and pharmacokinetics. In: Nightingale CH, Murakawa T, Ambrose PG, Marcel Dekker AG (eds) Microbiology and pharmacokinetics. CRC Press, Basel

[CR29] Olechnowicz J, Jaśkowski JM (2014). Mastitis in small ruminants. Med Weter.

[CR30] Owens WE, Watts JL (1987). Effects of milk on the activity of antimicrobials against *Staphylococcus aureus* isolated from bovine udders. J Dairy Sci.

[CR31] Papich MA, Riviere JE, Papich MG (2018). Chloramphenicol and derivatives, macrolides, lincosamides, and miscellaneous antimicrobials. Veterinary pharmacology & therapeutics.

[CR32] Persson Y, Olofsson I (2011). Direct and indirect measurement of somatic cell count as indicator of intramammary infection in dairy goats. Acta Vet Scand.

[CR33] Poumarat F, Gautier-Bouchardon AV, Bergonier D (2016). Diversity and variation in antimicrobial susceptibility patterns over time in *Mycoplasma agalactiae* isolates collected from sheep and goats in France. J Appl Microbiol.

[CR34] Pruul H, McDonald PJ (1992). Potentiation of azithromycin activity against *Escherichia coli* by human serum ultrafiltrate. J Antimicrob Ther.

[CR35] Rose M, Menge M, Bohland C (2013). Pharmacokinetics of tildipirosin in porcine plasma, lung tissue, and bronchial fluid and effects of test conditions on in vitro activity against reference strains and field isolates of *Actinobacillus pleuropneumoniae*. J Vet Pharmacol Ther.

[CR36] Ruegg PL (2017). A 100-year review: mastitis detection, management, and prevention. J Dairy Sci.

[CR37] Serrano-Rodríguez JM, Cárceles-García C, Cárceles-Rodríguez CM (2017). Susceptibility and PK/PD relationships of *Staphylococcus aureus* strains from ovine and caprine with clinical mastitis against five veterinary fluoroquinolones. Vet Rec.

[CR38] Shinozuka Y, Kawai K, Takeda A (2019). Influence of oxytetracycline susceptibility as a first-line antibiotic on the clinical outcome in dairy cattle with acute *Escherichia coli* mastitis. J Vet Med Sci.

[CR39] Stepanić V, Žiher D, Gabelica-Marković V, Jelić D, Nunhuck S, Valko K, Koštrun S (2012). Physicochemical profile of macrolides and their comparison with small molecules. Eur J Med Chem.

[CR40] Thomas V, De Jong A, Moyaert H (2015). Antimicrobial susceptibility monitoring of mastitis pathogens isolated from acute cases of clinical mastitis in dairy cows across Europe VetPath Results. Int J Antimicrob Agents.

[CR41] Virdis S, Scarano C, Cossu F (2010). Antibiotic resistance in *Staphylococcus aureus* and coagulase negative staphylococci isolated from goats with subclinical mastitis. Vet Med Int.

[CR42] Zarogoulidis P, Papanas N, Kioumis I (2012). Macrolides: from in vitro anti-inflammatory and immunomodulatory properties to clinical practice in respiratory diseases. Eur J Clin Pharmacol.

[CR43] Zhou YF, Bu MX, Liu P, Sun J, Liu YH, Liao XP (2020). Epidemiological and PK/PD cutoff values determination and PK/PD-based dose assessment of gamithromycin against *Haemophilus parasuis* in piglets. BMC Vet Res.

